# In Response

**DOI:** 10.4269/ajtmh.2012.12-0291b

**Published:** 2012-09-05

**Authors:** Donald S. Shepard, Wu Zeng, Peter Amico, Angelique K. Rwiyereka, Carlos Avila-Figueroa

**Affiliations:** Schneider Institutes for Health Policy; Heller School, Brandeis University; Waltham, Massachusetts; E-mails: shepard@brandeis.edu, wuzengcn@brandeis.edu, pamico@brandeis.edu, and gasugi@gmail.com; Abt Associates; Bethesda, Maryland; E-mail: carlos_avila@abtassoc.com

Dear Sir:

We thank Fan and others^1^ for their questions about potential bias in the selection of health centers (HCs) that received human immunodeficiency virus/acquired immunodeficiency syndrome (HIV/AIDS) funding and their controls.

Our study used a three-layered approach to minimize the risk of confounding and bias. First, we achieved exact matching on all the variables available at the central level (ownership, phase of performance-based financing, and district income). Second, in the course of our site visits, we collected data on contextual variables that might affect outcomes (coverage of community-based health insurance, bus accessibility, and background of the director) and controlled for these data in our multivariable regressions. Finally, we based our analysis on started logs, including random effects model specification and data from 2002 on all facilities prior to their initiation of antiretroviral therapy (ART). Differences on unobserved characteristics are possible in any observational study. However, this analytical approach, based on the panel data, controlled for any remaining differences between HIV/AIDS and control HCs.

Fan and others^1^ speculated that political connections may have been a factor in the assignment of HIV/AIDS services. We found no evidence of such political interference. This finding is consistent with the country's relatively high ranking on government effectiveness, which is noted in a blog by Fan and others.^2^

A strength of our study design is this multivariable specification. Similar to difference in differences, it does not require that the HIV/AIDS and control HCs be exactly comparable in baseline characteristics; therefore, we did not perform the matching tests described in the letter by Fan and others.^1^ Rather, our specification assumes only that, in the absence of the initiation of ART services, the rates of growth in primary care services in the two groups of facilities would have been comparable. Our finding of similar or higher growth rates for primary care services HIV/AIDS HCs means that such facilities with higher baseline values tended to achieve higher absolute increases in these services than control HCs.

In conclusion, we feel that our regression specification makes the risk of bias very unlikely and that this study helps to generate useful evidence from observational data.
